# Metal tolerance and Cd phytoremoval ability in *Pisum sativum* grown in spiked nutrient solution

**DOI:** 10.1007/s10265-023-01493-1

**Published:** 2023-09-07

**Authors:** Edith Cruzado-Tafur, Aleksandra Orzoł, Adrian Gołębiowski, Paweł Pomastowski, Mateusz Cichorek, Jacek Olszewski, Justyna Walczak-Skierska, Bogusław Buszewski, Małgorzata Szultka-Młyńska, Katarzyna Głowacka

**Affiliations:** 1 Department of Plant Physiology, Genetics and Biotechnology, University of Warmia and Mazury in Olsztyn, Oczapowskiego 1a, 10-719 Olsztyn, Poland; 2 Department of Environmental Chemistry and Bioanalytics, Faculty of Chemistry, Nicolaus Copernicus University, Gagarin 7, 87-100 Torun, Poland; 3 Centre for Modern Interdisciplinary Technologies, Nicolaus Copernicus University, Wilenska 4, 87-100 Torun, Poland; 4 Experimental Education Unit, University of Warmia and Mazury in Olsztyn, Plac Łódzki 1, 10-721 Olsztyn, Poland

**Keywords:** Bioaccumulation factor, Cadmium, Metal tolerance, Phytoremoval ability, *Pisum sativum* L., Translocation factor

## Abstract

In the presented study, the effects of cadmium (Cd) stress and silicon (Si) supplementation on the pea plant (*Pisum sativum* L.) were investigated. The tendency to accumulate cadmium in the relevant morphological parts of the plant (roots and shoots respectively)—bioaccumulation, the transfer of this element in the plant (translocation) and the physiological parameters of the plant through indicators of oxidative stress were determined. Model studies were carried out at pH values 6.0 and 5.0 plant growth conditions in the hydroponic cultivation. It was shown that Cd accumulates mostly in plant roots at both pH levels. However, the Cd content is higher in the plants grown at lower pH. The Cd translocation factor was below 1.0, which indicates that the pea is an excluder plant. The contamination of the plant growth environment with Cd causes the increased antioxidant stress by the growing parameters of the total phenolic content (TPC), polyphenol oxidase activity (PPO), the malondialdehyde (MDA) and lipid peroxidation (LP). The results obtained showed that the supplementation with Si reduces these parameters, thus lowering the oxidative stress of the plant. Moreover, supplementation with Si leads to a lower content of Cd in the roots and reduces bioaccumulation of Cd in shoots and roots of pea plants.

## Introduction

Plants display diverse types of interactions with several environmental factors. In addition, plants are constantly confronted with biotic and abiotic stresses, which induce disruptions in their metabolism, leading to physiological, molecular and cellular adaptations, which may reduce their productivity (Rejeb et al. 2014). Heavy metals are considered to be an important stress factor for plants, causing harmful effects on cellular and physiological processes, with the negative impact of these elements being highly dependent on their concentration, while the uptake of these elements is influenced by abiotic and biotic conditions (Fryzova et al. 2018). Cadmium (Cd) is considered to be one of the most phytotoxic heavy metals, and because it is easily taken up by plants, this is the main entry pathway into the food chain (Tiwari and Lata 2018). Cd uptake and accumulation may lead to severe toxicity to plants, thus threatening their growth and development (Zhou et al. 2022). Furthermore, Cd raises a particular concern because it can also accumulate in high quantities in leaves, where its content can be 10–500 times higher than in plants grown in unpolluted environments (Huang et al. 2020). Also, Cd can severely alter activities of several enzymes, resulting in stunted growth, leaf epinasty, chlorosis, alterations in chloroplast ultrastructure, inhibition of photosynthesis, pollen germination and distortion of pollen tube growth, induction of lipid peroxidation, and disruption of antioxidant systems (Tiwari and Lata 2018). Depending on the plant species, plants can accumulate distinct amounts of Cd, which can also vary depending on the Cd concentration in the rooting medium (Wang et al. 2018). Moreover, Cd absorption and translocation can vary greatly, even among cultivars or genotypes of the same species (Pietrini et al. 2009). Some plant species thrive in the presence of high concentrations of metals (termed metallophytes) by limiting the metal uptake or the translocation to the shoots (excluders), or by storing metals in their shoots (accumulators) (Godinho et al. 2018). Plants have evolved barriers in the roots to prevent Cd short distance transport, i.e. from cell to cell, and long distance transport, i.e. from root to shoot (Lux et al. 2011). The anatomical/apoplastic barriers such as maturation of the exodermis and endodermis developed earlier and more extensively in maize plant roots grown in aeroponics rather than in hydroponics (Redjala et al. 2011). The formation of apoplastic barriers such as the increased formation of suberin lamellae in the endodermis after Cd treatment were observed also in pea plants grown in perlite (Głowacka et al. 2019). The apoplastic pathway plays an important role in root-shoot Cd translocation in rice (Qi et al. 2020). Qi et al. (2020) observed deposition of apoplastic barriers closer to the root tip and increased amounts of lignin and suberin in low Cd-accumulating cultivars. This is an indicator that the radial transport of Cd in the root is crucial to the reduction of Cd root-to-shoot translocation (Anwen et al. 2021; Qi et al. 2020).

It is known that Cd taken up by plants causes oxidative damage in cells, which results in lipid peroxidation. Malondialdehyde (MDA), a marker of the lipid oxidation process in plants, indicates peroxidation processes, which cause oxidative modifications in the cell wall (Manquián-Cerda et al. 2016). Cd induces the production of reactive oxygen species (ROS) while inhibiting enzymatic activity, protein denaturation, and non-functionalization of the lipid membrane. In consequence, as part of the defence system, the plant produces antioxidant metabolites, such as glutathione and polyphenols (Haider et al. 2022). It has been shown that Cd interferes with plants’ uptake, transport and use of several elements (i.e., Ca, Mg, P, and K) along with water (Das et al. 1997; Głowacka et al. 2022). The effect of Cd in plants depends on its concentration in the soil. It has been shown that when the concentration of Cd in soil increases, the plant growth and number of leaves on a plant decrease. Therefore, physiological and biochemical processes occurring in plants depend on the content of Cd, and genetic traits are responsible for plants’ sensitivity to Cd (Jawad Hassan et al. 2020). Moreover, it was shown that the use of Cd in the cultivation of aquatic plants (*Pistia stratiotes*) induced the production of MDA in roots and leaves, and increased the activity of antioxidant enzymes. According to Li et al. (2013), peroxidase and superoxide dismutase provide better protection against Cd-induced oxidative damage than catalase, which has lower activity in the analyzed tissues. A similar dependence of the increase in the activity of antioxidant enzymes in the pea crop treated with Cd was shown by El-Okkiah et al. (2022). The toxic effect of Cd was limited by the supplementation of plants with Si, which reduced the content of MDA in the examined tissues.

On the other hand, the use of silicon (Si) to improve plant production has been widely discussed in recent years since Si decreases the uptake of some toxic elements, makes plants more tolerant to salinity, and increases the biomass of food crops (Greger et al. 2018). The function of Si is to protect the plant from various abiotic and biotic stresses or else alleviate their adverse effects (Ma and Yamaji 2008). Plant roots uptake Si in the form H_4_SiO_4_ (known as dissolved silicate) (Kabata-Pendias 2010), which is an uncharged monomeric molecule, when the solution pH is below 9 (Ma and Yamaji 2008). Si is deposited as SiO_2_ phytoliths in the lumen, cell walls and intercellular spaces (Pavlovic et al. 2021). Si accumulation in the plant tissues varies highly, for instance the Si concentration in plant shoots ranges from 0.1 to 10% Si in dry weight, depending on the plant species (Ma and Yamaji 2008).

One mechanism of Cd detoxification in the plants is the reduction of Cd uptake in their tissues, and because Si reduces the translocation of Cd from the root (through vacuolar sequestration) to aerial parts of plants, it can therefore prevent the adverse effect of Cd on photosynthesis (Kabir et al. 2016). Si-mediated Cd toxicity mitigation includes the formation of Cd–Si co-complexation in the cell wall reducing Cd transport to plant tissues, increased nutrient uptake, and decreased Cd uptake and translocation (El-Okkiah et al. 2022). The studies related to the Si-mediated reduction of metal stress in plants are carried out on a short-term basis and in hydroponic cultures (Rizwan et al. 2012). Hydroponics involves growing plants in a nutrient solution other than soil, and the majority of hydroponic cultures are maintained in controlled environments (generally, in greenhouses), with different support systems and water distribution technologies (Zajkowski and Short 2021). The efficient growth of plants through hydroponics is the purest form of growing crops owing to its controlled environmental factors and exclusion of pesticides (Haddad and Abahri 2022). The Cd taken up by plants affects the absorption, aggregation, and transport of micronutrients and macronutrients in plants (Bertoli et al. 2012), which are related to molecular relationships in the channels involved in the uptake of nutrients or plant metal transporters (Ismael et al. 2018). In order to understand the Si effects, availability, and accumulation on nutrient uptake, it is important to distinguish between studies made in soil and those made in hydroponics. Greger et al. (2018) reported studies of Si influence on different types of plants (maize, lettuce, pea, carrot, and wheat) that were grown in a hydroponic culture in a medium solution at pH 6.5, where Si increased the availability of the translocation of Mg, Ca, S, Mn, and Mo to shoots; for Fe, Cu, and Zn, this translocation decreased; and for K, P, N, Cl, and B—it was unaffected. Other studies have demonstrated that Si provides tolerance against the negative effects of Cd translocation in pea plants (*Pisum sativum* L.—Fabaceae family), alleviating Cd-induced pea growth and avoiding the yield reduction in soils (El-Okkiah et al. 2022). Also, Batool et al. (2022) showed that Cd stress significantly reduced growth in peas, but an application of Si enhanced the development of stressed plants by modulating the growth of fresh and dry biomass, improving the chlorophyll contents. In addition, the Cd taken up by plants reduced gas exchange, while the Si supplementation of the cowpea (*Vigna unguiculata*) crop caused the mitigation of its negative impact. Moreover, Si prevented the destruction of cell membranes caused by the toxic effects of Cd, and has also been shown to benefit the metabolism in cowpea roots growing in the soil. Cd-stress was also determined to cause increased production of ROS in cells, the level of which was correlated with the increased content of MDA (Pereira et al. 2018). Similar results were obtained by Rahman et al. (2017), who showed cellular membrane integrity and stable protein content in the roots and shoots of pea plants which were Si supplemented and Cd-treated in a hydroponic culture. However, Cd taken up by plants increased the content of phenolic compounds in the roots and aerial parts of the bean. These compounds are part of the non-enzymatic antioxidant system and save cells from oxidative damage by scavenging ROS (Benhabiles Ait El Hocine et al. 2020).

Thus, more studies are needed to understand the mobility of Cd in plants in controlled conditions (hydroponics), and also to recognize the implications of Si-mediated metal tolerance in controlled environments, which still remain unknown. The aim of the present work was to evaluate the Cd accumulation in tissues of *P. sativum*, the ability to uptake Cd to the shoots (BCF—bioaccumulation factor), and the transport of Cd within plant (TF translocation factor—the ratio of the metal concentration in the shoots to that in the roots), in a hydroponic culture conducted for 42 days, and to investigate the effect of Si supplementation provided to the plants in order to reduce the Cd toxicity, taking into account the influence of pH values of the medium solution. Furthermore, the parameters of oxidative stress were investigated. Thus, we studied: (1) to uptake and accumulation of Cd in the tissues of *P. sativum*, (2) the influence of nutrient solution (hydroponic) with different pH values on the bioconcentration and translocation Cd, (3) the effects of Si on the reduction of the Cd toxicity, and (4) the biochemical response (total phenolic content, polyphenol oxidase activity, and the malondialdehyde) of *P. sativum* under Si supplementation and Cd treatments.

## Experimental section

### Experiment design

The seeds of *P. sativum*, Pegaz cultivar were germinated in the darkness at 25 °C for 48 h (NÜVE ES 120 Incubator). Prior to the germination, the seeds were disinfected with isopropanol for 1 min and 2% sodium hypochlorite for 15 min. At the end of the disinfection process, the seeds were rinsed five times with demineralized and sterile water. The seedlings were transferred to sterile glass containers with 20 mL of half strength Hoagland solution (Table 1) at pH 6.0 for 6 days. The uniform pea seedlings were transferred to 1-L plastic containers (φ = 12 cm, four plants per container). The growth of the seedlings was continued at the half strength Hoagland’s solution for 7 days and followed with the full-strength Hoagland’s solution for the next 7 days. The 3-week-old plants were treated with 1 mM Na_2_SiO_3_ or 2 mM Na_2_SiO_3_ (Si) and/or 50 μM CdSO_4_ (Cd). The control plants were grown in the Hoagland solution without Cd and Si at pH 6.0. The cultivation was conducted for another 3 weeks in the Hoagland solution at pH 6.0. The nutrient solutions were replaced during the experiments every 2–3 days per week. The plants were grown in the incubation chamber (Snijders Scientific^™^ Plant Growth Cabinet) under 70% relative humidity at 25/20 °C and 10 h/14 h during the day and night, respectively. The plants were harvested to analyze the total phenolic, polyphenol oxidases (PPO) activity, malondialdehyde (MDA) content, and histochemical distribution of MDA. Plant tissues (shoots and roots) were lyophilized to analyze cadmium content and determine the bioaccumulation and translocation factors. Plants grown in the pH 5.0 Hoagland solution were also prepared for these analyzes.

**Table 1:** Composition of Hoagland solution

Compound	Concentration [mg L^−1^]
Macronutrients	
KNO_3_	505.5
Ca(NO_3_)_2_·4 H_2_O	1180.8
MgSO_4_·7 H_2_O	493.0
KH_2_PO_4_	136.1
Micronutrients	
MnCl_2_·2 H_2_O	1.81
ZnSO_4_·7 H_2_O	0.22
H_3_BO_3_	2.86
CuSO_4_·5 H_2_O	0.08
H_2_MoO_4_·H_2_O	0.09
C_10_H_12_N_2_NaFeO_8_	9.2

*Table 1 lists the quantification of the components of the Hoagland complete medium. The half-concentrated medium contains half as much of the indicated micronutrients and macronutrients

### Analytical methods

#### Total Cd content in plant

The vegetal material was dried and separated in organs. Dry samples were crushed and ground. 0.05 g of foliar samples were digested using 3 mL of the nitric acid (70%) and 1 mL of the hydrogen peroxide (30%) in microwave-assisted conditions at 180 °C for 20 min. At the end of the digestion, the container was allowed to cool, and the content was added to 10 mL with ultrapure water (18.2MΩ.cm). The metal determination was analysed by the ICP mass spectrometry (ICP-MS), Shimadzu 2,030 (Kyoto, Japan), while the experimental conditions of the analysis were carried out according to Orzoł et al. (2022). The standard addition was applied as a calibration method to minimize the matrix effect and to obtain the concentration of Cd in the samples. Standard solutions of Cd were prepared from their respective concentration of stock solutions, using as spike volume 100 μL of 100 μg L^–1^ of the Cd standard solution (standard addition method).

#### Bioaccumulation factor and translocation factor

The capacity of *P. sativum* to accumulate Cd in the hydroponic samples was evaluated based on the Cd uptake, the bioaccumulation factor (BCF), and the translocation factor (TF). BCF is defined as the ratio of the total metal concentration in shoots to that in the spiked solution, which is the ability to transport and uptake metals to the shoots (Jitar et al. 2015; Pietrini et al. 2013; Wang et al. 2018). TF is defined as the ratio of the metal concentration in the shoots to that in the roots (Pietrini et al. 2013; Wang et al. 2018).

#### Total phenolic content determination

The total phenolic contents were analysed spectrophotometrically using the Folin-Ciocalteu method (Singleton et al. 1999) with some modifications. The plant tissues (shoots and roots, 200 mg) were homogenized on ice in 2 mL of 80% ethanol. The homogenates were centrifuged for 20 min at 10 000 rpm at 4 °C. The supernatants were saved, and the residues were re-extracted in 80% ethanol. After centrifuging, two ethanolic extracts (supernatants) were pooled together and evaporated at room temperature. The dry residues were dissolved in water. Reagent was added to 1 mL of the sample (100 µL extract with 900 µL 0.9% NaCl), 0.5 mL of Folin-Ciocalteu’s (1:4). After 3 min, 4 mL of 20% Na_2_CO_3_ solution was added to the mixture and incubated in boiling water for 1 min. Afterwards, the sample was cooled, and the absorbance was measured with a spectrophotometer (Tecan Infinite 200 PRO) at λ = 650 nm against a blank without extract. The outcome data were expressed as μg catechol per mg dry weight.

#### Polyphenol oxidases activity quantification

The polyphenol oxidase activity was analyzed spectrophotometrically according to the Haplin and Lee (1987) method with some modifications. The plant tissues (shoots and roots, 200 mg) were homogenized on ice in 1 mL of 0.1 M sodium phosphate buffer (pH 6.5) with 1% polyvinylpyrrolidone (PVP). The homogenates were centrifuged for 10 min at 10 000 × *g* at 4 °C, and the supernatants were collected. The reaction mixture was prepared 30 min before determining the enzyme activity and contained 0.1 M sodium phosphate buffer (pH 6.5) along with catechol dissolved in McIlvaine buffer (pH 6.5). The enzyme extract (25 µL) was added to the reaction mixture (225 µL) and mixed thoroughly. The absorbance was measured with a spectrophotometer (Tecan Infinite 200 PRO) at λ = 420 nm. One unit [U] of PPO activity was expressed as a change of absorbance per minute per mg dry weight. The Bradford method was applied to determine the total content of protein (Bradford 1976).

#### Determination of malondialdehyde content

The malondialdehyde contents were determined spectrophotometrically according to the Dhindsa et al. (1981) method. The plant tissues (shoots and roots, 200 mg) were homogenized on ice in 1 mL of 0.1% trichloroacetic acid (TCA). The homogenates were centrifuged for 5 min at 10 000 × *g* at 4 °C. The supernatants were collected. The reaction mixture contained 400 µL of 0.5% thiobarbituric acid (TBA) prepared in 20% TCA. The enzyme extract (100 µL) was added to the reaction mixture, mixed thoroughly, and incubated at 95 °C for 30 min in a thermoblock (ThermoStat plus, Eppendorf). Afterwards, the sample was cooled in ice for 5 min, and the absorbance was measured with a spectrophotometer (Tecan Infinite 200 PRO) at λ = 532 nm and λ = 600 nm (non-specific absorbance) against a blank without extract. MDA concentrations were determined using the extinction coefficient 155 mM^−1^ × cm^−1^.

#### Histochemical distribution of MDA

The histochemical distribution of MDA was visualized by Schiff’s reagent according to the Pompella et al. (1987) method with some modifications. The plant tissues (leaves and roots) were washed in distilled water and placed in falcon tubes with 10% Schiff reagent for 10 min in the vacuum infiltration. Next, the containers with plant tissues were incubated for 1 h at room temperature in darkness. Leaves and roots were washed in distilled water and transferred to falcon tubes with 0.5% sodium metabisulfite solution in 0.05 M HCl for 2 min. Subsequently, plant tissues were washed with distilled water and placed in a storage solution (glycerol:ethanol; 1:4, *v/v*) to be photographed. Results were analyzed with Keyence microscopy (VHX Digital Microscope).

#### Statistical analysis

A two-way analysis of variance (ANOVA) was performed to determine differences between groups, followed by Tukey’s test with the significance level set at *p* ≤ 0.01. The statistical analysis was carried out using STATISTICA (ver. 13.1 Dell Inc. Tulsa, OK, USA).

## Results and discussion

### Cadmium concentrations in plant tissues

**Fig. 1:**
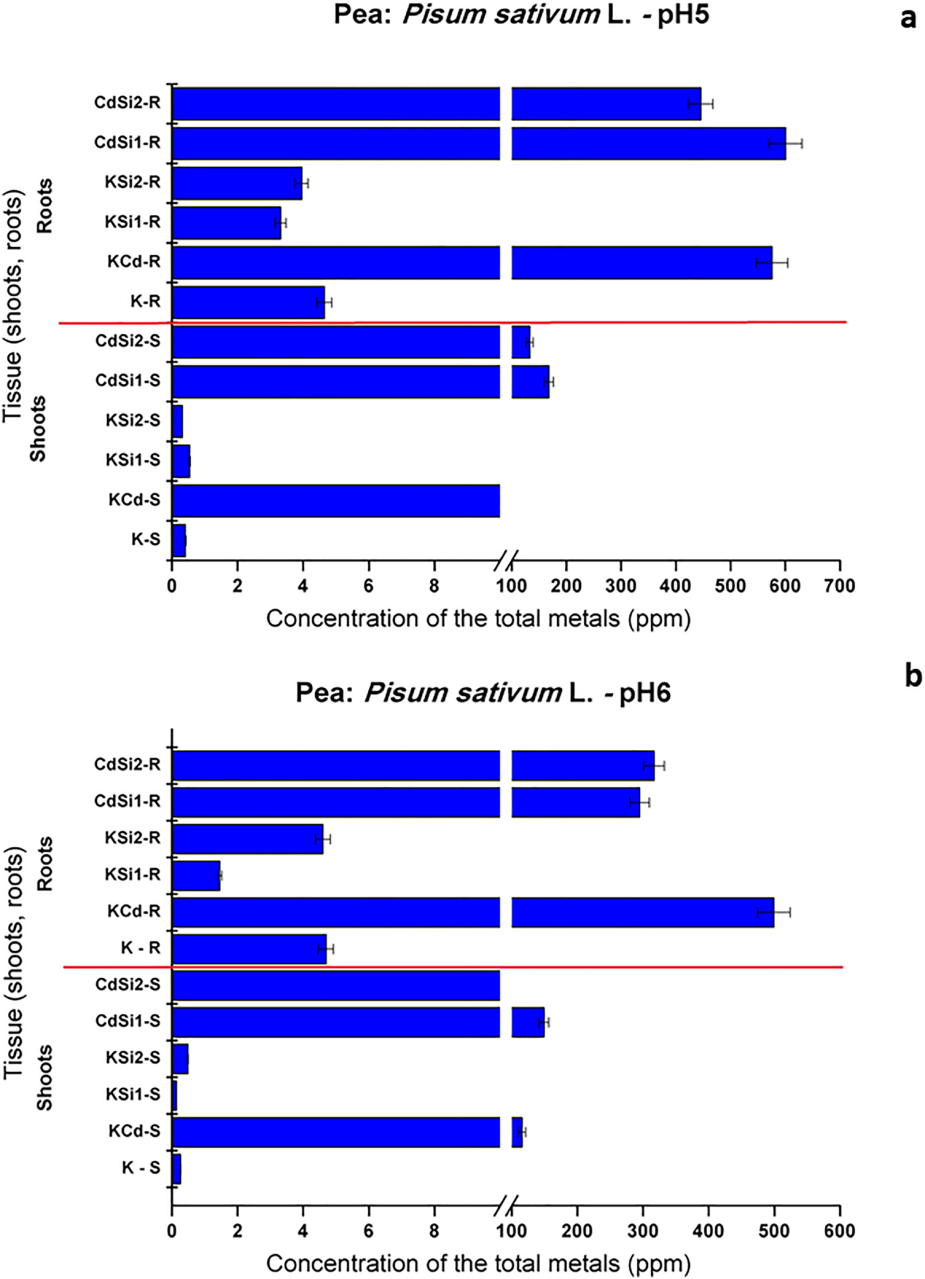
Cadmium content of *P. sativum* in solution **a** pH 5.0 and **b** pH 6.0

As depicted in Fig. 1, under various treatment applications to different pH, the application of Cd and Si affected the uptake and distribution of the metal in *P. sativum*. The concentration changes in all parts of *P. sativum* manifested the same trend for pH 5.0 and pH 6.0, showing an increased concentration with the increasing amounts of the spiked metal solution (50 µM CdSO_4_ and Si medium).

The Cd content increased in the whole plant under Cd stress, showing a higher Cd content in roots than in shoots. Cd concentrations in roots and shoots were higher for KCd, CdSi1, and CdSi2 for both pH 5.0 and pH 6.0. Roots showed a greater amount of Cd concentrations than shoots. For pH 5.0, the concentrations in roots amounted 564, 597, and 431 mg kg^−1^ for KCd, CdSi1, and CdSi2 respectively; for pH 6.0, Cd concentrations in roots showed 456, 292, and 315 mg kg^−1^ for KCd, CdSi1, and CdSi2. Metal mobility within plant tissues is governed by pH, the oxidation–reduction state, competing cations, hydrolysis, polymerization, and formation of insoluble salts (Kabata-Pendias 2010). Through pH, metal availability can also be modified by plant roots, the regulation of which affects the plant metal uptake (Cruzado-Tafur et al. 2021). The exposure to 50 µM Cd of the pea plants reflected a quick response to the presence of a high Cd concentration (Hernández et al. 1997), in roots and shoots. *P. sativum* tends to retain Cd in the roots, while the longer exposure times allow Cd to be translocated to shoots, without displaying any noticeable phytotoxic effects (Gusmão Lima et al. 2006).

On the other hand, the control samples (K) showed similar behaviour to the samples in the silicon solution (KSi1, and KSi2), with low concentrations of Cd in shoots and roots for both pHs. The application of Si along with Cd did not stop the Cd accumulation in shoots and roots (CdSi1 and CdSi2), as compared with Cd stressed sample. When samples were treated solely with Si, plants showed the Cd content similar to that of the control plants. Cd concentrations for the treatments KCd, CdSi1, and CdSi2 in both pH, the threshold value in aerial parts of 0.2 mg kg^−1^ considered sufficient, and also, exceeded the values of 30 mg kg^−1^ considered excessive or toxic. The pea accumulated in shoots and roots evinced significant values of Cd, the excessive presence of this metal inhibited the growth of the plants (Orzoł et al. 2022).

It is important to understand the concentration and distribution proportion of Cd in the root tissues of the studied cultivars in order to find out the tolerant and sensitive pea cultivars in the different treatments. Cd is toxic to plants even at low concentrations; it affects the morphological, physiological, biochemical, and molecular levels; with respect to physiological disorders, it increases the oxidative stress (causing the overproduction of reactive oxygen species [ROS]) (El Rasafi et al. 2022). The obtained results revealed a higher accumulation of Cd in the root than in the shoot, which is consistent with the findings of Ullah et al. (2020) in *Cicer arietinum* belonging to the Fabaceae family. The accumulation of Cd in the root and the shoot of the examined pea cultivars was strongly related to the concentration-dependence. *P. sativum*, the plant species of our study is a plant belonging to the Fabaceae family, which showed a significant elevation in the oxidative indices, as measured in terms of lipid peroxidation (Anjum et al. 2014). The trace element concentrations in plants depend highly on their growth media as nutrient solutions, the absorption of which is related to the concentrations in the solutions, plant species, and the development stage. The accumulation of some ions can take place against a concentration gradient (Kabata-Pendias 2010). It is important to underline that the hydroponics culture allows the control of the Cd concentrations bioavailability but it is quite different from the environmental conditions (Gusmão Lima et al. 2006).

The obtained results are in harmony with the results of other authors (Delpérée and Lutts 2008; Ullah et al. 2020), which demonstrated that Cd has a negative effect on plant water relations. It was observed that a limited supply of water decreased the uptake and the accumulation of metals; also, certain tolerant cultivars accumulate high Cd in the shoot, which is the distinguishing feature of plants having high biomass. The foliar uptake consists of two phases, nonmetabolic cuticular penetration (major route of entry), and metabolic mechanisms (responsible for transporting ions). Trace elements taken up by leaves can be translocated to other plant tissues, depending on the plant organ, its age, and the element involved (Kabata-Pendias 2010).

The Cd content in the pea and its distribution proportion can be used as parameters to detect stress tolerance in the treatment’s solutions. Hence, KCd was sensitive because higher Cd contents were observed in their root for both pHs. This may affect the cell wall of the root, nutritional uptake and signaling mechanism of metabolites leading to less above-ground biomass (Ullah et al. 2020). In contrast, KSi1 and KSi2 had low values for such parameters showing more sensitivity under Cd stress in the silicon presence.

### Bioconcentration factor (BCF) in *P. sativum*

**Fig. 2:**
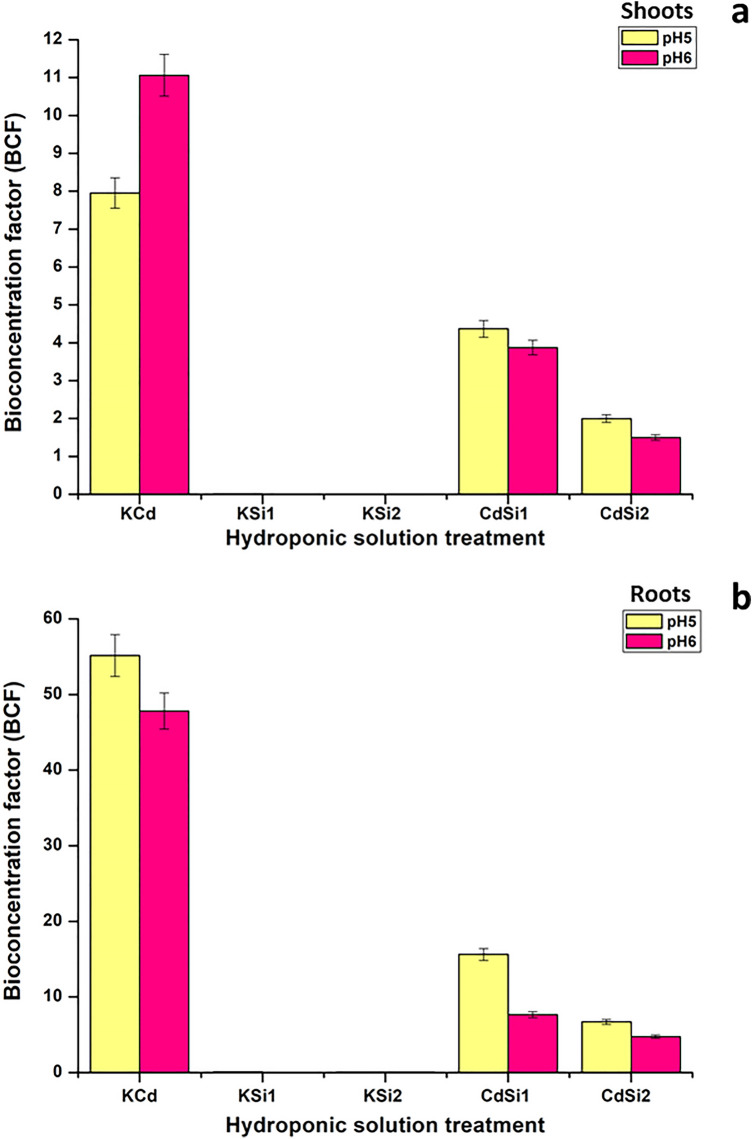
Bioconcentration factor (BCF) values of Cd for pH 5.0 and pH 6.0 hydroponic solution in the cultivation of *P. sativum*, **a** shoots, **b** roots

To express the ability of a plant to accumulate metals against a concentration gradient, the bioconcentration factor (BCF) is a useful parameter for evaluating this potential (Diwan et al. 2010). Depending on the specific ability of plants and the metal uptake between plant species, the following uptake characteristics can be distinguished such as accumulation, indication, and exclusion (Kabata-Pendias 2010). The BCF was calculated just for 5 treatments: KCd (50 µM CdSO_4_), KSi1 (1mM Si medium), KSi2 (2 mM Si medium), CdSi1 (Cd and 1 mM Si medium), CdSi2 (Cd and 2 mM Si medium), because with these treatments the plant is subjected to a strong stress of the metal content. Consequently, samples with the hydroponic solution in pH 5.0 (yellow bars) and pH 6.0 (pink bars) for shoots and roots (Fig. 2), showed the same behaviour. KCd, CdSi1 and CdSi2 samples showed a BCF higher than 1, high BCF for Cd; also, the values with these treatments were increased, which indicated that these samples uptake more metal in the areal parts of the plants. For KSi1 and KSi2, the BCF were < 1 for both pHs, which could be due to the presence of silicon, without cadmium stress. Moreover, these results are in accordance with the determination of the total metal concentration.

The peak BCF value for Cd in the hydroponic solution for shoots was 11 by pH 6.0, followed of 8 by pH 5.0 (Fig. 2a) at the concentration of 50 µM of Cd. For the roots (Fig. 2b) it was 48 by pH 6.0 and 55 by pH 5.0. This was the case possibly, because of the fact that Cd is a natural mobile element easily absorbed by roots thus causing high accumulation in the plant (Syuhaida et al. 2014). On the other hand, this result indicates that the Cd accumulation rate decreases with the presence of silicon as in the samples of CdSi1 and Cd Si2 at the addition of 1 mM and 2 mM of Si, the values of which were 4 and 3 for shoots respectively, and 16 and 8 for roots (pH 5.0 and pH 6.0). The order of Cd tolerance in the hydroponic solutions in both pH were KCd > CdSi1 > CdSi2 > KSi1 > KSi2.

Plants during their evolution and course of life, developed several biochemical mechanisms that resulted in the adaptation and tolerance of trace elements and new environments (Kabata-Pendias 2010). Since Cd is not essential for plant growth, it may cause toxicity and reduction in the plant growth (Syuhaida et al. 2014). The foliar uptake consists of the nonmetabolic cuticular penetration (major route of entry), and metabolic mechanisms (account for element accumulation against a concentration gradient); the second one is responsible for transporting ions across the plasma membrane and into the cell protoplast (Kabata-Pendias 2010).

Similar results were reported by Galal et al. (2021), where *P. sativum* had Cd high accumulation power. For this reason, the examined BCF was higher than 1, which is in accordance with our finding that the hydroponic solution under Cd stress had the highest BCF. Some researchers (Badora 2002) reported that silicon can reduce the toxic action towards the growth of such plants as millet, soybean, barley, but not rice, cotton, wheat or pea. This could explain that the addition of silicon to the hydroponic solution failed to stop the uptake of Cd in the aerial parts of the plant. Ullah et al. (2020), showed that the BCF of chickpea (*Cicer arietinum*, family: Fabaceae) was dose-dependent of the nutrient solution and went up significantly with the increase of Cd stress, which is an agreement with our results. The BCF constitutes an excellent indicator of metal accumulation because it takes into account the ratio of the Cd concentration in the growth medium and plant tissues (root and shoot, respectively) (Liu et al. 2009).

### Translocation factor (TF) of *P. sativum* in hydroponic solution

**Fig. 3:**
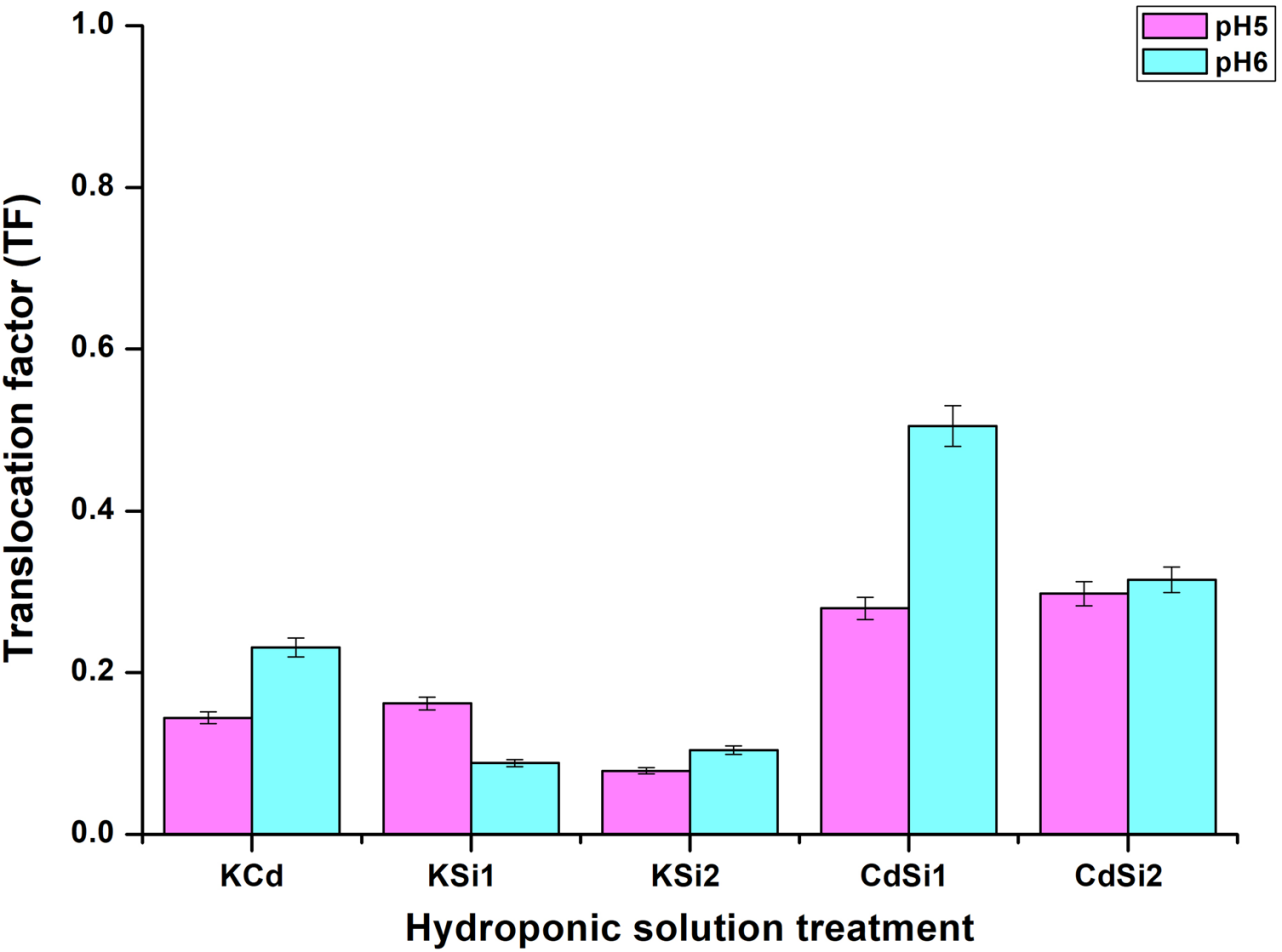
Translocation factor (TF) values of Cd in *P. sativum*

Many processes are involved in the transport of ions within plant tissues and organs. They allow the control of cation translocation in plants, such as the movement in xylem, movement in phloem, storage, accumulation, and immobilization (Kabata-Pendias 2010). The translocation factor (TF) was calculated along with the BCF only for five treatments: KCd (50 µM CdSO_4_), KSi1 (1mM Si medium), KSi2 (2 mM Si medium), CdSi1 (Cd and 1 mM Si medium), CdSi2 (Cd and 2 mM Si medium) (Fig. 3).

The results showed that the TF values for both pHs (pH 5.0: purple bars, pH 6.0: sky blue bars) were variables in each sample of the hydroponic solution. The treatments of KCd (0.2), CdSi1 (0.5) and CdSi2 (0.3) showed higher values; however, these values were lower than one. In addition, all the samples showed TF < 1. Taking into account the results of the BCF, which in the case of KCd, CdSi1, and CdSi2 samples were higher than one, with the TF results being lower than one, our results show the phytostabilization potential. However, they are excluders, because they possess mechanisms that maintain the low metal uptake (Gajić et al. 2020) in this case cadmium-hydroponic solution and small shoot metal contents. For the samples KSi1 and KSi2, with the BCF < 1 and the TF < 1 they are also considered excluders. For both pH values, the TF values were around 0.1. These very low values could be due to the concentration of Si in the medium without cadmium stress and to the binding to silicates within plant tissues, which restricted the translocation (Greger et al. 2018). Excluders are plants that do not react with an excess in the content of an element in their tissues (Khusnidinov et al. 2020). Therefore, *P. sativum* in all the treatments can be considered to be a cadmium excluder plant.

The immobilization of metals in the roots could result from various processes like the complexationes of cations with organic acids (e.g., citric, malic, and amino acids) preventing the immobilization in the xylem and allowing their transfer to the shoots, taking a dominating impact on the translocation of metals to the above-ground parts (Kabata-Pendias 2010). Sterckeman and Thomine (2020) reported that *P. sativum* cultivated and exposed to Cd in hydroponics, showed good Cd levels in the cell-wall fraction of leaves and roots, but the ratio sharply decreased after reaching a peak, as if the root retention capacities became overloaded. Galal et al. (2021) *P. sativum* reported low TF values (0.49 and 0.58, respectively), which coincides with the results of this study. On the other hand, numerous other factors such as pH, the oxidation–reduction state, hydrolysis, polymerization, competing cations, and the formation of insoluble salts, affect the metal mobility within plant tissues (Kabata-Pendias 2010).

### Cd stress indicators in the plant

**Fig. 4:**
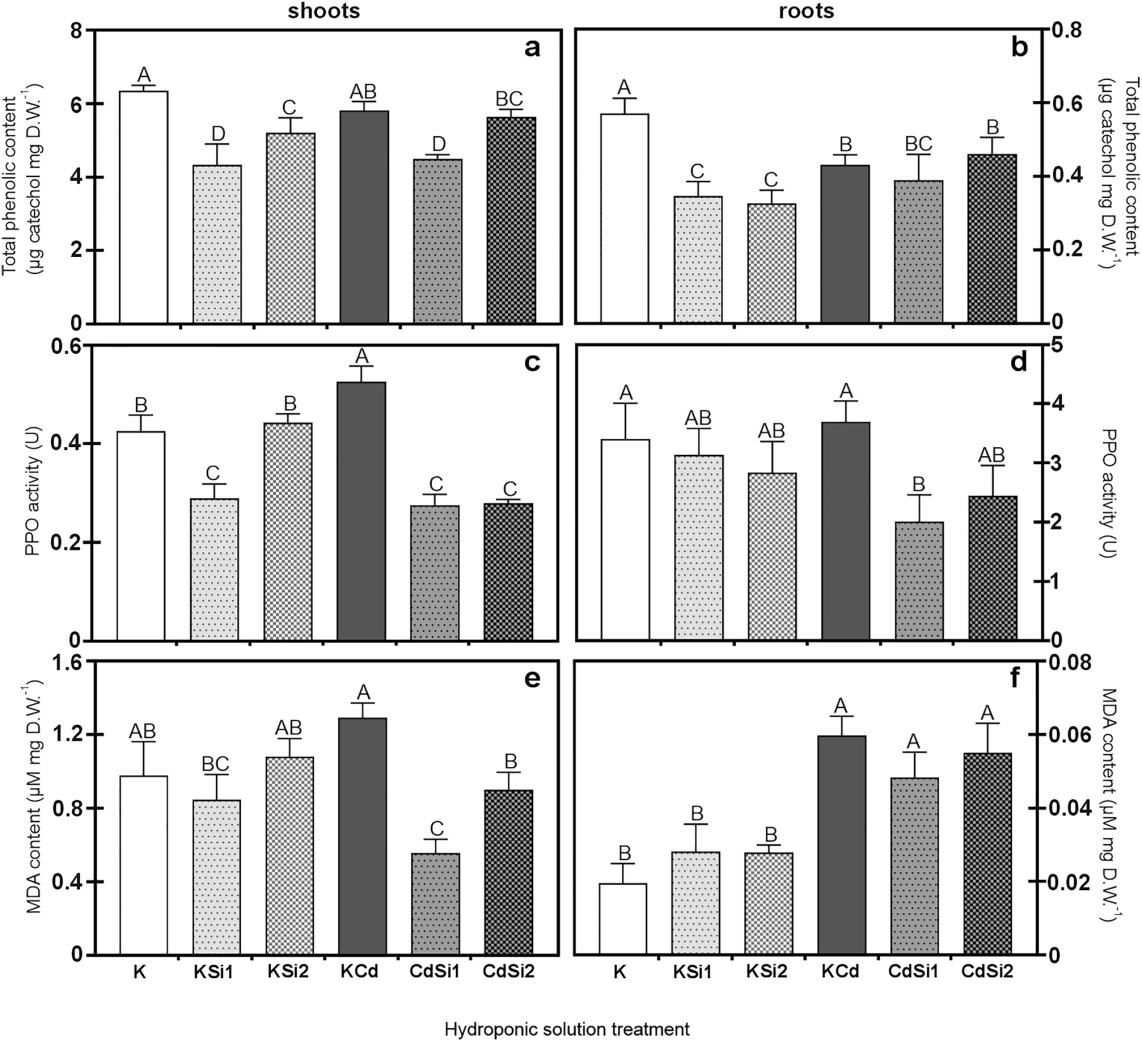
The total phenolic contents in shoots (**a**) and roots (**b**), the polyphenol oxidase activity (PPO) in shoots (**c**) and roots (**d**) and the malondialdehyde (MDA) content in shoots (**e**) and roots (**f**) of *P. sativum* 3 weeks after Si-supplementation (1 mM Na_2_SiO_3_ or 2 mM Na_2_SiO_3_) and/or Cd-treatment (50 µM CdSO_4_) (*n* = 4). Each value is the means of four replicates ± SD. Different letters represent significant differences (*p* ≤ 0.01)

Plant phenolic compounds are important constituents of defense responses and can act as antioxidants. The total phenolic contents (Fig. 4a, b) in shoots and roots of pea plants grown in the control Hoagland solution and supplemented with 1 mM Si or 2 mM Si and/or treated 50 μM Cd were estimated. The total content of phenolics in the shoots and roots of the plants treated with Cd was significantly lower than in the control plants. The phenolic content in plants is correlated with the activity of PPO (Fig. 4c, d), the increased activity of which may cause the oxidation of phenols to quinones. The reduced phenolic content was also observed in the shoots and roots of plants supplemented with Si compared to the shoots and roots of the control plants. Whereas using 1 mM and 2 mM Si supplementation significantly decreased the phenolic in the shoots and roots compared to control plants. It has also been shown that the application of Cd and Si 1 mM in cultivation significantly decreased the phenolic content in plant tissues compared to the Cd-treated plants’ phenolic content. Elguera et al. (2013) observed that total phenolic content tended to decrease in leaves with the increasing concentration of Cd applied to the cultivation of *Lepidium sativum*. In addition, the total phenolic content in the leaves of tomatoes treated with heavy metals (Cd, Cu and Pb) was also significantly lower than the control plants’ leaves. The increase in the phenolic content was visible after the plants were treated with the highest concentration of Pb (50 ppm), and the phenolic content was comparable to that in the control plants (Kisa et al. 2019). A slight increase in the total phenolic content was also observed in *Erica andevalensis* leaves treated with various concentrations of Cd. The authors expected an increase in the phenolic content in the leaves of plants treated with the highest concentration of Cd (50 μg Cd g soil^−1^). The reduction in the content of phenolic compounds may be caused by a disturbance of the reaction of the antioxidant system using the phenolic due to a too high concentration of Cd. In their opinion, the synthesis of phenoxy radicals could also occur. Phenoxy radicals acting as prooxidants, disturb the induction of the antioxidant reaction. The synthesis and release of phenolic are then reduced (Márquez-García et al. 2012). Part of the research on total phenolic content showed that heavy metal stress significantly increased the total phenolic in plants. The phenolic content in the Al-treated plants was higher than in the control plants. The simultaneous Si supplementation and Al treatment reduced the total phenolic content of the analyzed plants. The Si application reduces the effects of heavy metal stress in plants. The authors also point out that the total content of phenolic is related to the content of flavonoid compounds and antioxidant enzymes (Dar et al. 2022).

Plants treated with Cd showed significantly increased activity of polyphenol oxidase in the shoots compared to control plants. Meanwhile, a decrease in the activity of PPO as compared to the shoots of control and Cd-treated plants was observed in plants supplemented with Si. Reduced enzyme activity was observed in the roots of plants supplemented with Si. A simultaneous treatment of plants with Cd and their supplementation with 1 mM and 2 mM Si significantly decreased the PPO activity in the shoots and the roots compared to Cd-treated plants. The lower enzyme activity in the shoots of plants supplemented with 1 mM and 2 mM Si and treated with Cd compared to the Cd-treated plants is comparable to the PPO activity in the shoots supplemented with 1 mM Si. This may indicate a positive effect of the Si supplementation in plants, especially since the activity of this enzyme in the roots of plants treated with Cd and supplemented with 1 mM and 2 mM Si was lower than the activity of PPO in the roots of plants treated with Cd. The PPO is a terminal oxidase that directly passes electrons to O_2_ during the oxidation of intermediates from the plant respiration. Along with other enzymes, PPO can also participate in synthesizing compounds containing phenolic groups, such as lignin (Thipyapong et al. 2007). The activity of PPO is related to the radical coupling of monolignols to form lignin and flavonoid polymerization in the cell wall. The oxidase activity in plant tissues increases, among others, during cadmium stress (Sullivan 2015). Zheng et al. (2010) demonstrated an increase in the PPO activity in licorice cotyledons and radicles. A significant increase in the enzyme activity was observed in tissues treated with Cd at concentrations of 0.4, 0.1 and 0.2 mM L^−1^. However, the activity decreased significantly in hypocotyls in higher concentrations of Cd (0.2 and 0.4 mM L Cd^−1^) by 24.2% and 33.3%, respectively. An increase in the PPO activity was also observed in *Arabidopsis thaliana* leaves. An increase in the enzyme activity by 20% in the cultivation (compared to the leaves of the control plants) after using 100 µM Cd was observed (Saffar et al. 2008). The PPO activity might be related to its function in the phenolic compound synthesis, which plays an important role in detoxifying heavy metals in plants (Ruiz et al. 1999). The effect of Si on the activity of PPO under abiotic stress was analyzed by Abdelaal et al. (2020). The activity of PPO was significantly increased in salt-stressed (3,000 ppm concentration) treated leaves of plants as compared to stressed and Si-supplemented leaves of plants and control leaves by 19% and 50%, respectively. A decrease in the enzymatic activity may result from the fact that Si has an important role in regulating the stability of the plasma membrane in the plant cells (Kučerová et al. 2020).

The malondialdehyde (MDA) content as the lipid peroxidation (LPO) index in pea plants under Si supplemented and/or Cd-treated was also established (Fig. 4e, f). The increased content of MDA in plant tissues is an indication of the overproduction of reactive oxygen species. The results showed that the MDA content increased in Cd-treated plants compared to control plants. The highest significant content increase was in the roots of plants treated with only Cd and supplemented Si and Cd-treated plants compared to the roots of control plants. The overall MDA content of the shoots was higher than that of the roots of pea plants. A significant increase in the MDA content was also observed in the shoots, where the amount of MDA was 0.98 µM mg^−1^ at the control, and a noticeable increase of 1.29 µM mg^−1^ was recorded in Cd-treated plants. At the same time, the MDA content of the shoots of plants that grew with Si 1 mM or 2 mM was significantly different from this parameter in the control shoot plants. A decrease in the MDA content was observed in the shoots of plants supplemented Si (1 mM and 2 mM) and Cd-treated, where the amount of the MDA reached 0.56 and 0.90 µM mg^−1^, at Si 1mM and Si 2 mM, respectively. The reduced content of the MDA in these tissues, as compared to the Cd-treated shoots of plants, confirms a positive effect of Si on the reduction of oxidative damage to biological membranes. The MDA is a biomarker of oxidative stress and is one of the final products of LPO in the cells (Del Rio et al. 2005). Pereira et al. (2018) analyzed cowpea plants (*Vigna unguiculata*) under Cd stress and indicated a correlation between ROS and MDA content in plant tissues. They showed that the increased MDA content depends on the increased level of ROS in the cells (Pereira et al. 2018). The effect of Si on MDA content (and thus the stability of the membranes) was also assessed under Al stress. The authors showed the increased content of MDA in leaves and roots treated with Al (by 26% and 28.2%, respectively). They found that a simultaneous supplementation of Si plants reduces the leakage of electrolytes from the analyzed tissues. This means that Si impacts the stability of biological membranes in plants exposed to Al stress (Shen et al. 2014).

**Fig. 5:**
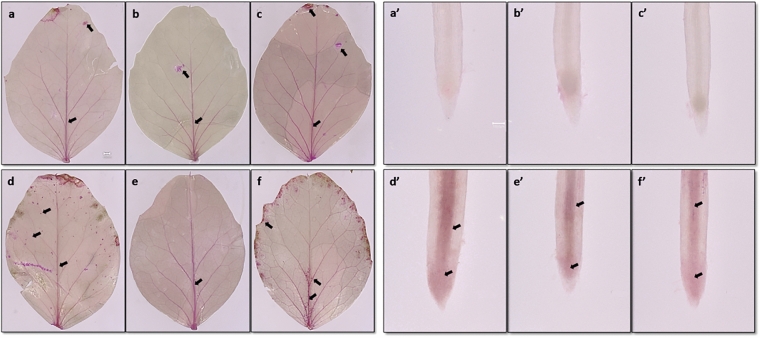
Detection of lipid peroxidation using Schiff’s reagent in *P. sativum* leaves (scale bar = 1 mm) and roots (scale bar = 100 μm) three weeks after Si-supplemented (1 mM or 2 mM Na_2_SiO_3_) and/or Cd-treated (50 µM CdSO_4_): **a**, **a**’ control; **b**, **b**’ Si 1 mM; **c**, **c**’ Si 2 mM; **d**, **d**’ Cd; **e**, **e**’ Cd and Si 1 mM; **f**, **f**’ Cd and Si 2 mM

The histochemical staining with Schiff’s reagent confirmed Cd-induced oxidative damage in cells of leaves and roots (Fig. 5). The applied staining highlights the LPO of the membranes through an intense red coloration. In the control plants, the faint red color was localized at the veins in the leaves and vascular bundles in the roots. The same color was observed in Si-supplemented (1 mM and 2 mM) plant tissues (Fig. 5b, c, b’, c’). In Si-supplemented roots, the appearance of the meristem zone was more visible. The Cd treatment increased the LPO at the leaves and roots of pea plants (Fig. 5d, d’). The leaves of the Cd-treated plants showed a red color of the veins and intense colored areas all over their surface compared to the control plants. In addition, the area near the leaf petiole had a more intense color. A similar visualization of LPO was observed in the leaves of plants supplemented with 2 mM Si and treated with Cd (Fig. 5f). At the same time, the leaves of the plants supplemented with a lower concentration of Si (1 mM) and treated with Cd were similar to the control leaves. The Cd-induced increase in the LPO level was observed in the roots, even in plants supplemented with Si (Fig. 5d’, e’, f’). The increased color intensity of the leaves and roots of the Cd-treated plants indicates a disturbance of the integrity of the cell membranes. Schiff’s reagent, used in the analysis, visualized LPO in plant tissues (Yamamoto et al. 2001), detecting a marker of oxidative stress related to the free radical process of lipid oxidation (MDA). The induction of LPO in the cell membranes of Cd-treated plants was also demonstrated by Piacentini et al. (2020) by analyzing, among other things, the effect of Cd (100 μM CdSO_4_) on rice roots. The authors showed the induction of ROS formation after the treatment of Cd plants and demonstrated the presence of LPO in the cell membranes of the Cd-treated plants. The red color of the control roots was significantly lower than that of these plants. In contrast, the supplementation with Si plants regulates the accumulation of ROS and LPO. According to the authors, the Si supplementation of plants reduced butachlor’s toxic effects. On the other hand, using Si in the rice cultivation reduced oxidative stress and improved the plant growth and development. The presented LPO visualization confirmed the positive impact of Si in cultivating plants exposed to the selective pre-emergence herbicide (Tripthi et al. 2020). Also, LPO in plants exposed to water stress was limited in plants supplemented with Si. The histochemical analysis of tomato roots was conducted 6 days after water stress, and it was indicated that LPO is significantly reduced in plants supplemented with Si (Shi et al. 2016).

## Conclusion

The present study showed that *P. sativum* accumulated Cd in tissues, mainly in roots and less in shoots, but the supplementation of Si played a critical role in withstanding Cd toxicity and restoring the normal growth and development of the plant; besides, the mobility of the metal was also governed by pH of the nutrient solution, hence the Cd content was higher in the plants grown at lower pH. The mechanisms counteracting Cd toxicity in roots to reduce the bioaccumulation in shoots could be related to the concentration of Si in the hydroponic solution or to the restricted Cd translocation via binding to silicates within plant tissues, because the translocation factor was below 1, showing that *P. sativum* has a phytostabilization potential and a Cd excluder behaviour. Also, it is possible to suggest that Si addition enhances the translocation of transport molecules, which could contribute to the metal transport from root to shoot, diminishing the deficiency of the plant (Greger et al. 2018). In addition, it can be concluded that the Si application reduces the toxicity effect of Cd stress on plants. The exogenous Si application stimulates the production of total phenolics, and affects the defence PPO activities under heavy metal stress. Additionally, the supplementation of the pea plants with Si reduces the content of MDA, one of final lipid peroxidation products in cells, in the shoots and roots. Therefore, it can be asserted that Si maintains the stability of biological membranes in plants exposed to Cd stress. The changes in the analyzed parameters may indicate that Si induces stress tolerance in pea plants.

## Data Availability

The data that supports the findings of this study are available in this article.
